# Engineered Cementitious Composites with Super-Sulfated Cement: Mechanical, Physical, and Durability Performance

**DOI:** 10.3390/ma17102240

**Published:** 2024-05-10

**Authors:** Shahin Zokaei, Hocine Siad, Mohamed Lachemi, Obaid Mahmoodi, Emircan Ozcelikci, Mustafa Şahmaran

**Affiliations:** 1 Department of Civil Engineering, Toronto Metropolitan University, Toronto, ON M5B 2K3, Canada; shahin.zokaei@torontomu.ca (S.Z.); mlachemi@torontomu.ca (M.L.); omahmoodi@torontomu.ca (O.M.); 2 Department of Civil Engineering, Hacettepe University, Ankara 06800, Türkiye; emirozcelikci@gmail.com (E.O.); sahmaran@hacettepe.edu.tr (M.Ş.)

**Keywords:** super-sulphated cement (SSC), engineered cementitious composites (ECCs), fly ash, shrinkage, ductility

## Abstract

This study aimed to bridge a research gap by exploring the utilization of super-sulphated cement (SSC) in engineered cementitious composites (ECCs) as a sustainable alternative to ordinary Portland cement (OPC)-based mixtures. The SSC was designed with slag, gypsum, and a small amount of OPC. The primary objective was to investigate the effects of incorporating SSC, both with and without fly ash (FA), at various FA/SSC ratios between 0 and 1.5. A comprehensive evaluation was conducted to assess the performance of the ECC-SSC mixtures, including the compressive and flexural strengths, ductility, ultrasonic pulse velocity, rapid chloride permeability, and drying shrinkage. Additionally, advanced microstructural evaluation techniques such as scanning electron microscopy (SEM) with energy dispersive X-ray (EDX) analysis as well as X-ray diffraction (XRD) analysis were employed to analyze the reaction products in selected mixtures. The results showed that the ECC mixture produced with SSC exhibited comparable strength to the ECC-OPC. In general, all the SSC-based ECCs fulfilled the criteria for various engineering applications, especially when the fly ash to SSC ratios were 0 and 0.8. In addition, ECCs with FA/SSC ratios of 1.2 and 1.5 showed ultra-ductile performance higher than the control ECC. Interestingly, all the FA-based ECC-SSC presented lower shrinkage characteristics than the control OPC-based ECC.

## 1. Introduction

Engineered cementitious composites (ECCs) have gained significant attention for their exceptional tensile strain capacity, making them a desirable choice for constructing earthquake-resistant structures and carrying out repairs and retrofits [1]. The incorporation of microfibers has played a crucial role in enhancing ECCs’ performance by improving their ductility and enabling the formation of multiple micro-cracks under load. Despite their remarkable properties, ECCs face challenges associated with their high reliance on ordinary Portland cement (OPC), leading to significant environmental and economic concerns [2]. To tackle these challenges, researchers have been exploring the use of various waste materials, such as silica fume, slag, and class C or F fly ash as partial replacements for OPC in ECC-mix designs [3,4]. In addition, studies are now exploring alternative design strategies that aim to minimize or completely eliminate the need for OPC, considering its substantial contribution (6–10%) to global anthropogenic CO_2_ emissions [5,6].

As a suitable alternative to OPC, super-sulphated cement (SSC) is a slag-based binding material made of more than 80% ground granulated blast furnace slag (GGBFS), 10–20% anhydrite, and low amounts of alkali activator (mostly OPC). Consequently, SSC has been the subject of much research in recent years due to its ability to reduce the environmental impact of cementitious materials [7]. In fact, by using SSC as a binder, the amount of CO_2_ emitted is shown to decrease by a minimum of 49% [8], since it uses a high amount of slag with minimal energy in its production. Furthermore, in order to produce SSC, the basic components are simply ground into powder of a specific fineness and uniformly blended, eliminating the need for calcination. This not only saves energy but also reduces the price of OPC concretes, up to 26% [9,10]. The advantages of SSC are also sulfate and alkali–silica reaction (ASR) resistance, a low heat of hydration, and the utilization of industrial byproducts from the steel manufacturing industry [11]. The hydration process of SSC was stated to depend on the influence of alkali and sulfate, which can regulate the reactivity of slag [12]. Ettringite (C_6_AS_3_H_32_) and calcium silicate hydrate (CSH) are formed as the reaction products from the slag reacting with calcium sulfate during SSC hydration. Thus, in SSC mixtures, ettringite as the principal hydration product is primarily responsible for its strength development at the early stages of hydration [13]. Although its slow early strength development resulted in the underutilization of SSC in the industry [14], the addition of OPC, with the outcome of the formation of calcium hydroxide (CH) in the matrix, was proven to create a suitable alkaline environment to promote the hardening properties of SSC matrices [15]. According to Guo et al. [16], during the mixing process, the slag dissolves in the alkaline environment, thereby releasing Ca^2+^ and Al^2+^ ions. The amount of ettringite formed is directly influenced by the pH level of the pore solution, and, in turn, this impacts the early strength of the hydrated cement paste. In situations where the pH level is excessively high or when there is a high concentration of alkaline activator, the SO_4_ content tends to be relatively low in the matrix. Under such conditions, ettringite can easily transform into monosulfate (AFm), while additional C-S-H and C-A-S-H phases could be formed. In addition, at an appropriate pH range, confirmed to be between 10.8 and 12.5 [17], the existing anhydrite provides adequate sulphate and SO_3_^2−^ ions, which react with the released Ca^2+^ and Al^2+^ ions to form ettringite (C_6_ASH_32_) [18]. Generally, the amount of alkali activator had a stronger effect on the hydration and strength development of SSC compared to the anhydrite content [19].

In addition to OPC and anhydrite, the generation of ettringite is also shown to be influenced by the chemical composition of the slag. Masoudi and Hooton [14] performed extensive research on SSC developments to reach better mechanical strengths. Low-alumina slag showed ettringite and C-S-H as the main hydration products of gypsum, while hydrotalcite was also observed, depending on the amount of available MgO in the slag. As a result, low-alumina slag required more alkali activation than high-alumina slag. Although high-alumina slags in concrete showed higher strength development at 7 days, they exhibited similar strengths to the low-alumina slag at 28 days. The greater early strengths of high-alumina slag SSC mortars were also confirmed by Da Luz and Hooton [20], who found that this was valid at all curing temperatures. The addition of supplementary cementitious materials such as fly ash (FA) was also investigated, with the circulating fluidized bed combustion FA as an activator [21]. Promising results were reported showing enhanced strengths at increased FA amounts from 10% to 30%, which was explained by the higher presence of alumina and associated C-A-S-H gel. Moreover, when incorporating FA alongside SSC replacements ranging from 15% to 50%, the initial mechanical strengths of the SSC concrete were inferior to those of OPC concrete. However, as time progressed, the results approached or surpassed those of OPC concrete, although this trend was less pronounced as the FA replacement ratio increased from 30% to 50% [22].

Despite the literature offering various attempts to optimize the composition and characteristics of SSC mixtures, the integration of SSC into ECC has remained unexplored until now, notwithstanding the potential sustainability enhancements associated with integrating SSC into ECC compositions. This gap of knowledge is particularly significant, considering the possible synergies between SSC and fly ash, as the typical supplementary material used in ECC. Thus, the primary aim of this research is to address this crucial research gap by examining the inclusion of SSC in ECC mixtures, with a focus on their mechanical, physical, and durability properties as compared to conventional ECC that uses OPC. This study specifically evaluates the influence of SSC, both with and without FA, on the compressive and flexural strengths, ductility, ultrasonic pulse velocity (UPV), chloride permeability (RCPT), and drying shrinkage characteristics of ECC-SSC mixtures. Different FA/SSC ratios of up to 1.5 were explored and extensively analyzed using SEM-EDS and X-ray diffraction (XRD) to gain a better understanding of the reaction kinetics and ECC product matrices. The anticipated results of this study will offer valuable insights into the feasibility and potential benefits of incorporating SSC into ECC formulations, thereby contributing to the development of more sustainable construction material.

## 2. Experimental Work

### 2.1. Materials

**Figure 1:**
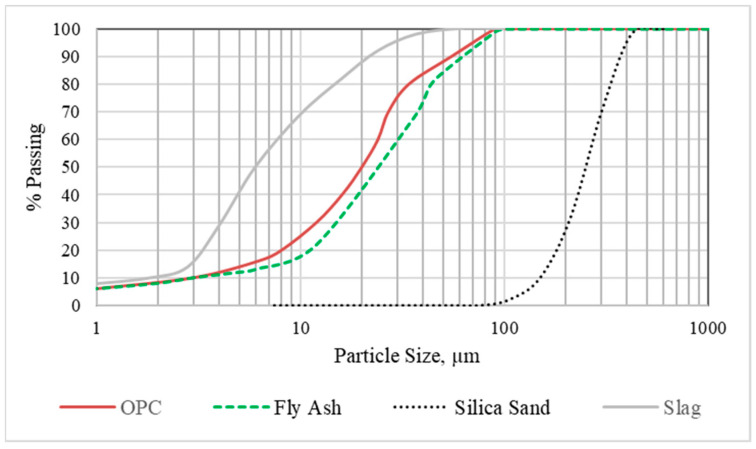
Particle size distribution of OPC, fly ash, and slag powders, as well as silica sand.

In this study, ordinary Portland cement (OPC), class-F fly ash (FA), silica sand with a maximum particle size and specific gravity of 400 μm and 2.65, respectively, and polyvinyl alcohol (PVA) fibers measuring 8 mm in length and 38 μm in diameter were utilized in the control mixture. For super-sulphated cement (SSC)-based mixtures, there is a wide variation in formulations in the literature, regarding the optimum mixture composition. Many researchers have reported that SSC consists of 80–85% slag, 10–15% anhydrite, and around 5% clinker. However, the Al_2_O_3_ content of slag has to be considered since it plays an essential role in influencing the mechanical performance of SSC composition. According to Masoudi and Hooton [14], slags with lower CaO and Al_2_O_3_ content necessitate at least 8% OPC for their activation, while at higher CaO and Al_2_O_3_, slags require lower alkali content. A preliminary study was conducted to find the optimum composition based on the slag used in this study, which was characterized with a CaO + Al_2_O_3_ amount of 46.8% (Table 1). Three different mortar mixtures were cast by varying the amounts of slag, gypsum, and OPC, while preserving constant sand/OPC and water/OPC ratios at 3 and 0.5, respectively. Table 2 depicts the amounts of slag, gypsum, and clinker in each composition as compared to the standard mortar with 100% OPC. As can be seen in Table 2, SSC incorporating 85% slag, 10% OPC, and 5% gypsum achieved the highest compressive strength at 3 and 7 days. Thus, this composition was selected to be used in all the SSC-ECC mixtures. Except for the slag, the same OPC, gypsum, silica sand, and PVA fibers were utilized in the control and SSC-ECCs. The slag conformed to ASTM C989/989M standards [23]. The particle size distributions (PSD) of the constituent materials are presented in Figure 1.

### 2.2. ECC Compositions and Specimen Preparation

A normal ECC that comprised a typical FA/OPC ratio of 1.2 was chosen as the base mixture. To determine the optimum FA content in the ECC-SSC mixtures, different FA-to-SSC ratios (0, 0.8, 1.2, and 1.5) were utilized. Just as in the control ECC (ECC-CTL), the water-to-binder and sand-to-binder ratios were kept constant at 0.27 and 0.36, respectively, while polyvinyl alcohol (PVA) fibers were added at 2% of the total volume. Table 3 provides the detailed mix proportions of the materials utilized in the ECC-SSCs and ECC-CTL. Each ECC formulation was assigned a unique code that indicated the cement type and FA amount. For example, ECC-SSC-1.2F denotes the composition prepared with SSC at an FA-to-SSC ratio of 1.2.

In the experimental phase of the study, the solid materials were first mixed in a 120-liter capacity mixer for 1 min and 30 s. Then, 80% of the water was introduced into the mixture, and mixing continued for two more minutes. Afterwards, the remaining water was mixed with a high-range water-reducing admixture (HRWRA) and added to the mixture. Finally, PVA fibers were added to the mixture, and blending continued for another 3 min. The flow after casting was measured by means of the mini-cone flow table conforming to ASTM C230 in order to attain a targeted diameter of around 18 cm to 23 cm [24]. The amount of HRWRA required for each mixture is given in Table 2. As can be seen from Table 2, the amount of HRWRA decreased in the SSC mixtures where the fly ash content increased due to the positive effect of its particles on the workability thanks to its spherical structure [25,26]. After the fresh properties were evaluated, the mixtures were poured into molds of various sizes including: (50 × 50) mm cubes, (50 × 75 × 360) mm prisms, cylinders with 100 mm diameter and 200 mm depth, and (25 × 25 × 285) mm mortar bars. A polyethylene plastic sheet was placed over the molds, and they were left in a laboratory environment of approximately 23 ± 2 °C for 24 h before demolding.

### 2.3. Testing Methods

In order to evaluate the mechanical performance of the ECC-CTL and ECC-SSC specimens produced within the scope of the study, compressive and four-point flexural strength tests were performed after curing at 50% relative humidity and 23 °C for 7, 28, and 120 days, with at least three specimens for each test. The compressive strength test followed the ASTM C39 standard [27], and the flexural strength test followed the ASTM C1399/C1399M standard [28]. UPV tests were carried out at 28, 60, 90, and 120 days to evaluate the density and overall integrity of the produced mixtures. They were performed using prismatic specimens with dimensions of (50 × 75 × 360) mm, in accordance with the guidelines of the ASTM C597 standard [29]. The RCPT was completed to evaluate the resistance of the ECC mixtures to chloride ion penetration. In the RCPT test, Ø100 × 50 mm specimens were prepared and tested after curing periods of 28, 60, 90, and 120 days, in accordance with the ASTM C1202 standard [30]. In addition, drying shrinkage tests were performed on 25 × 25 × 285 mm bar specimens up to 120 days. The initial length of the mortar bars was measured after demolding and used as a reference to calculate the shrinkage at different curing ages. A digital length comparator was used to ensure accurate measurement of the drying shrinkage. Drying shrinkage tests were carried out in accordance with the ASTM C596 standard [31]. A JEOL JSM-6380LV scanning electron microscope (JEOL, Tokyo, Japan) with an imaging resolution of 3.0 nm was employed to examine the effect of SSC and FA on the microstructure of the ECC specimens. The analysis involved SEM-EDS investigations conducted on the core of specimens that had been cured in water for a 120-day period.

## 3. Results

### 3.1. Compressive Strength

**Figure 2:**
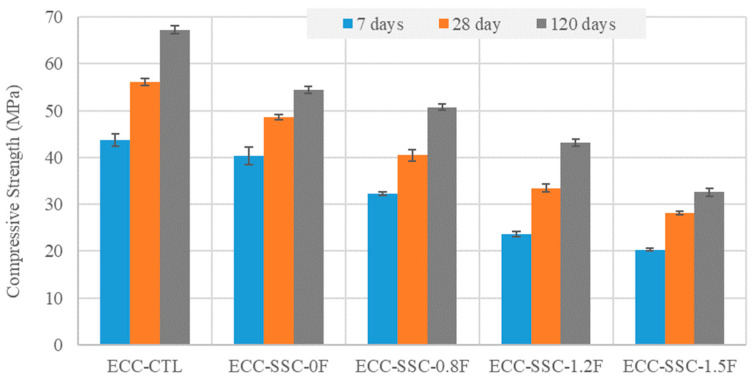
The compressive strength results of the mixtures.

The variation in the compressive strength of the SSC-based ECCs at 7, 28, and 120 days after initial casting is presented in Figure 2. In general, all SSC-based ECCs with different fly ash contents displayed lower compressive strengths than the control ECC at the curing ages between 7 and 120 days. However, at the early curing age of 7 days, ECC-SSC-0F showed almost the same result as the ECC-CTL, with a negligible difference of 1.4%. Thus, the pozzolanic properties and the high particle fineness (Figure 1) of the slag-blended SSC were useful for the compressive strength gain at the initial stages [14,32]. The results were clearly reduced with an increased fly ash amount in the SSC-ECCs. For instance, the compressive strength of ECC-CTL was 7.9, 26.3, 46.14, and 53.7% higher than that of the ECCs prepared with SSC at FA/SSC ratios of 0, 0.8, 1.2, and 1.5, sequentially, at 7 days. These differences were still high at later ages, with 13.4, 27.8, 40.4, and 49.9%, respectively, at 28 days, and 19.1, 24.7, 35.8, and 51.4%, respectively, at 120 days. It is known that the general hydration mechanism of SSC requires the alkali activator (OPC) to form calcium hydroxide from hydration reactions. This provides the required alkaline environment for the dissolution of the slag, releasing Ca^2+^ and Al^2+^ ions. The gypsum offers sulphate that reacts with these ions to form ettringite, as the main hydration product of SSC at the early curing ages [14]. Nevertheless, according to Sun et al. [33], the principal reaction product responsible for the later age strength development of SSC was mostly C-S-H rather than the ettringite-dependent early age strengths. Thus, in the case of the increased fly ash with SSC replacement, the lower alkali environment may have impacted the dissolution of both the slag and FA particles, leading to lower ettringite and C-S-H formation and decreased compressive strengths at higher FA contents. However, the pozzolanic activity of FA seems improved, with the advanced age results of the SSC-ECCs having lower differences than the ECC-CTL at 28 and 120 days, especially for ECC-SSC-1.2 F, where the difference compared to the ECC-CTL reduced from 46.14% at 7 days to 40.4% and 35.8% at 28 and 90 days, respectively. Interestingly, except for ECC-SSC-1.5 F, which presented lower strengths than 30 MPa at 28 days, all the other SSC-based ECCs satisfied the requirement of most engineering applications, especially at FA/SSC ratios of 0 and 0.8.

### 3.2. Flexural Strength

**Figure 3:**
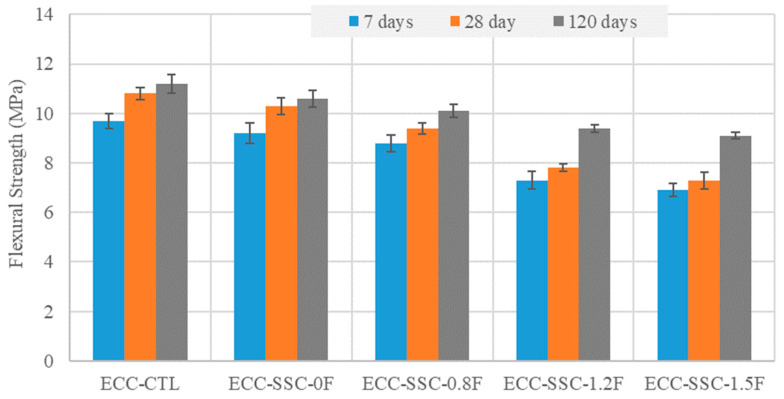
Effect of the SSC and FA on the flexural strengths of the ECC mixtures.

**Figure 4:**
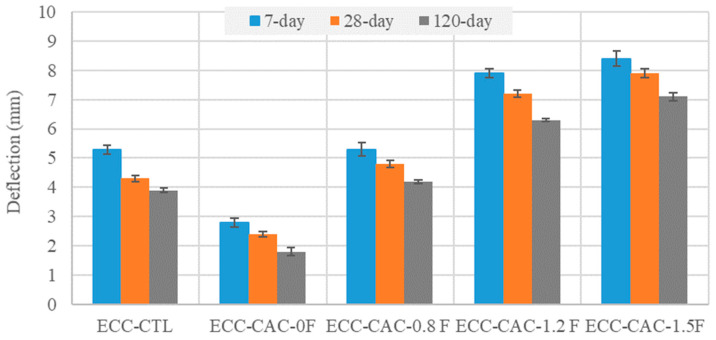
Effect of the SSC and FA on the deflections of the ECC mixtures.

The four-point flexural test was conducted to assess the flexural properties of the SSC-based ECC mixtures. The results of the flexural strengths and deflection capacities at failure are given in Figure 3 and Figure 4, respectively. From these figures, at all curing ages, the ultimate flexural strengths of all the SSC-based ECC specimens were lower compared to those of the ECC-CTL. For example, the flexural strength of the ECC-CTL was 4.9, 14.9, 38.5, and 48% higher than the SSC-based ECCs with FA/SSC ratios of 0, 0.8, 1.2, and 1.5, respectively, at 28 days. The ECC-SSC-0F exhibited the lowest flexural deformation values compared to the ECC-CTL and other SSC-ECCs at various curing ages. The possible reason for this reduction could be the micromechanical properties of SSC-ECC, in which the use of a high amount of slag particles may have decreased the potential for the higher ductility of the ECC, since slag has been shown to cause an increased fiber-to-matrix chemical bond as compared to the FA-based ECC-CTL [34]. This also agrees with the previous literature about the decreased ECC tensile strain, as the slag replacement level increased with the fly ash replacement [32], indicated to be mostly related to the limited slippage effect at the fiber interface pocket of the slag–ECC mixtures. However, the deformation values recorded for the SSC-ECCs in Figure 4 highly improved as the FA content was progressively increased from an FA/SSC ratio of 0.8 to 1.5. For instance, at 28 days, these mixtures exhibited deflections of up to 11.6, 67.4, and 83.7%, respectively, higher than that of the control ECC. The incorporation of a higher content of fly ash may have reduced the chemical bonding at the PVA fiber vicinities, leading to enhanced slippage ability at the fiber/matrix pocket. In addition, the spherical round shape and smooth surfaces of the many unreacted FA particles as compared to the reduced amount of angular- and irregular-shaped slag granules likely helped to decrease the matrix toughness and the related interface with FA particles, thus increasing the bending capability of the ECC [35,36,37].

### 3.3. Ultrasound Pulse Velocity

**Figure 5:**
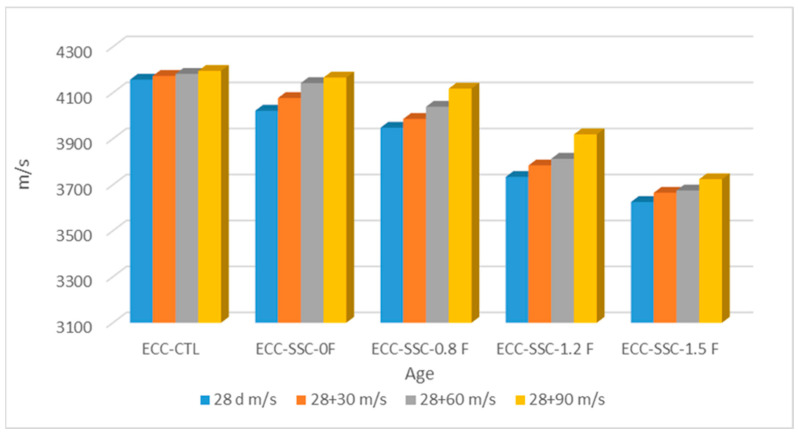
UPV results of sound SSC-based ECCs at different curing times.

The UPV values of the control and SSC-based ECCs at 28 days and up to 120 days after initial casting are displayed in Figure 5. According to this figure, all the SSC mixes obtained UPV values from 3625 to 4166 m/s at 28 days, which are under the category of good quality concretes with high compactness and reduced porosity [35]. Like the mechanical results, at all curing ages, lower UPV values were registered for the sound SSC-ECCs than the SSC-ECC. The UPV value of the ECC-CTL was 2.9, 4.8, 10.8, and 14.1% higher than the ECC-SSC-0 F, ECC-SSC-0.8 F, ECC-SSC-1.2 F and ECC-SSC-1.5 F, respectively, at 28 days. Furthermore, the use of FA in the SSC-based ECCs caused their UPV results to decease at all curing ages. However, at the end of the 28 + 90 days of moist curing, the differences between the ECC-CTL and the FA-based SSC-based ECCs appeared to be reduced compared to the initial measurements at 28 days. Taking as an example the UPV values for ECC-SSC-1.2 F, they increased from 3743 m/s at 28 days to 3919 m/s at 120 days, consequently decreasing their respective differences from 10.8 to 6.5% between 28 and 90 days when compared to the ECC-CTL. This is probably due to the improved reaction of the fly ash particles at later curing times; in particular, the lower volume of capillary pores at advanced ages of FA-SSC mixtures contributed to the system’s strength and impermeability, thus leading to enhanced UPV outcomes with longer curing periods [38].

### 3.4. Chloride Penetrability

**Figure 6:**
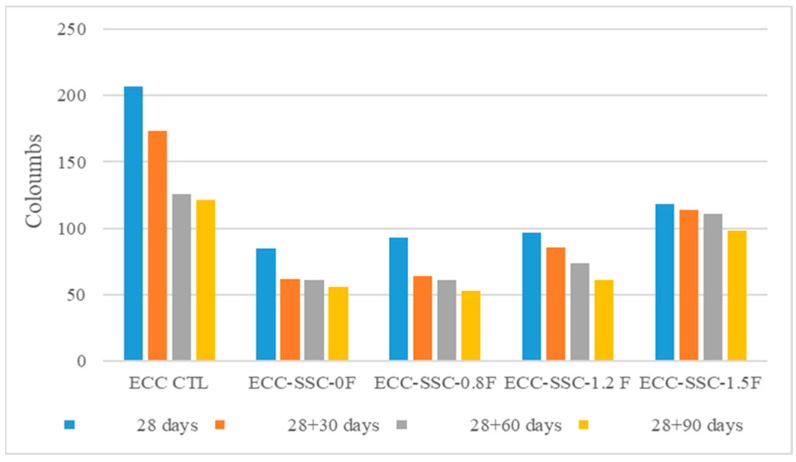
RCPT results of sound ECC-SSCs at different curing ages.

Figure 6 presents the RCPT values of the control and SSC-based ECCs. At all curing ages, except for ECC-SSC-1.5, the charge that passed through all the SSC–ECC mixtures was lower than 100 coulombs, indicating a negligible chloride permeability for these mixtures, according to ASTM C1202. In addition, the charge range for ECC-SSC-1.5 was slightly higher, ranging between 100 and 118 coulombs, suggesting that the concrete had significantly low to negligible chloride ion permeability. Compared to the ECC-CTL, all the sound SSC-based ECCs provided very low RCPT values at all ages. For example, the chloride ion penetrability of the SSC-ECCs with FA/SSC ratios of 0, 0.8, 1.2, and 1.5 were 59, 55, 53.1, and 43% lower than the control ECC at 28 days, respectively. The high reduction in the RCPT results with the use of SSC in normal concretes was also mentioned in a previous work by Masoudi [18]. According to this author, the lower charges passed through the SSC compositions are mostly due to their decreased pore solution conductivity as compared to OPC concretes. It is worth mentioning that the SSC system contains 80% slag, which may have significantly reduced the alkalinity of the pore solution and consequently impacted the conductivity of the ECCs, thus resulting in lower RCPTs than in the ECC-CTL. In addition, a portion of chloride may have been solidified by the ettringite-based reaction products of the SSC-ECCs, which additionally reduced the chloride penetrability of these mixtures. This is in line with the findings of Nguyen et al. [39], who showed that the ettringite products formed in SSC concrete were highly effective in bounding Cl− from sea water, proving their ability in neutralizing most of the available chloride, as an anti-corrosion concrete material. The slight resistance increments observed with the increased FA content also corroborate the earlier explanation regarding the significant chemical influence of the matrix solution on chloride binding. The higher FA content with SSC is expected to restrict ettringite formation [14,32], typically resulting in the reduced resistance of fly ash-based SSC-ECCs to chloride penetration. The pozzolanic reaction of fly ash can result in the creation of extra C-S-H phases that possess enhanced capability in capturing chloride ions. These newly generated C-S-H phases might have served as a physical obstruction and chemically immobilized chloride ions, diminishing their movement and infiltration into the concrete matrix [40]. Additionally, while a higher fly ash content may restrict ettringite formation, the overall advantages of these mechanisms typically surpass any potential downsides related to reduced ettringite, resulting in enhanced resistance to chloride. The provided alumina from fly ash is another reason that can be considered, since fly ash with a high alumina amount and different calcium contents showed generally higher chloride binding capacity than OPC [41].

### 3.5. Drying Shrinkage of SSC-Based ECCs

**Figure 7:**
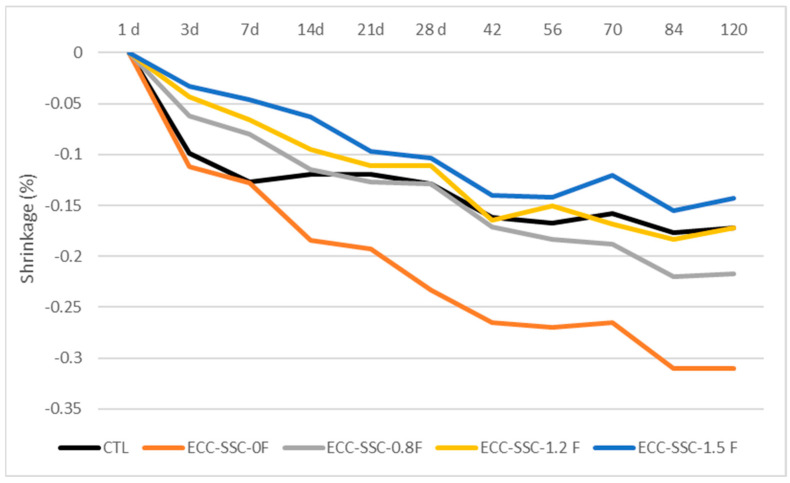
Drying shrinkage of the control and ECC-SSC mixtures.

Figure 7 shows the drying shrinkage results of ECCs with different fly ash-to-SSC cement ratios over a period of 120 days of curing at controlled 50% RH and 23 °C. The ECC-SSC-0F indicated higher shrinkage than the ECC-CTL starting the first week and continuing up to 120 days of testing. Nevertheless, lower shrinkage results were acquired when FA was introduced into the SSC-ECC mixes. This caused almost a similar trend of results between ECC-CTL and the ECC-SSC-0.8F and ECC-SSC-1.2F, though slight increments were noticed in the shrinkage values of the ECC-SSC-0.8F than the ECC-CTL at longer curing times. In addition, to some extent, the drying shrinkage of the ECC-SSC-1.5 F was lower compared to the ECC-CTL at all testing ages. For example, at 28 and 120 days, the shrinkage values of ECC-SSC-1.5 F were 19.7% and 17.2% lower than those of the ECC-CTL, respectively. The lower drying shrinkage for the mix with higher fly ash content could be due to the fact that the unreacted FA particles may have served as fine aggregates helping to restrain the shrinkage of the SSC-ECCs [42]. In addition, spherically shaped FA particles possibly introduced a denser particle packing that improved the pore distribution [32] and helped reduce the moisture evaporation from the matrix. This explanation is also supported by the higher differences observed between the FA-based SSC-ECCs and ECC-CTL at early curing ages up to 14 days of testing, which followed a reducing trend with the continued curing time. While at 7 days, ECC-SSC-0.8 F and ECC-SSC-1.2 F exhibited 36.5% and 47.9% lower shrinkage than ECC-CTL, these differences reduced to less than 1% and 13% at 28 days. Thus, the decreased amount and size of the FA granules at later ages may have reduced the filling effect of FA and its ability to retain a stable volume for cementitious materials [32]. Unlike FA-based SSC-ECCs, a higher drying shrinkage trend was registered for ECC-SSC-0F compared to the control ECC. This can be due to the larger water solution on the angular and irregularly shaped slag granules, because the dominant slag content in ECC-SSC-0F may have enhanced its internal moisture evaporation and shrinkage over the curing age [41,42].

### 3.6. SEM/EDX Analysis

**Figure 8:**
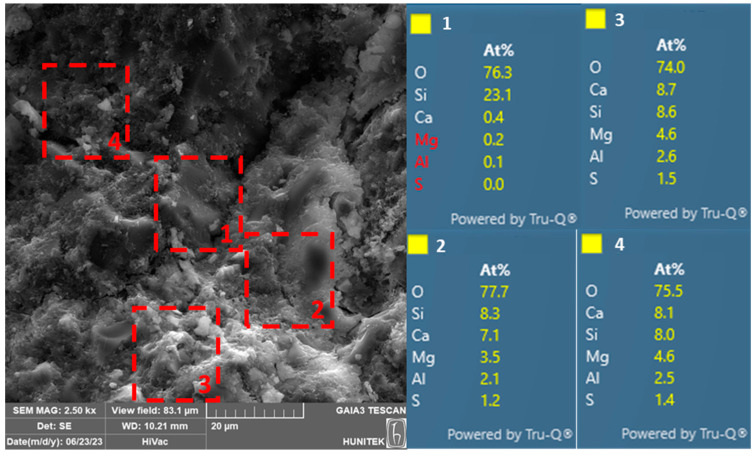
SEM/EDX of ECC-SSC-0F coded mixture after 120 d.

**Figure 9:**
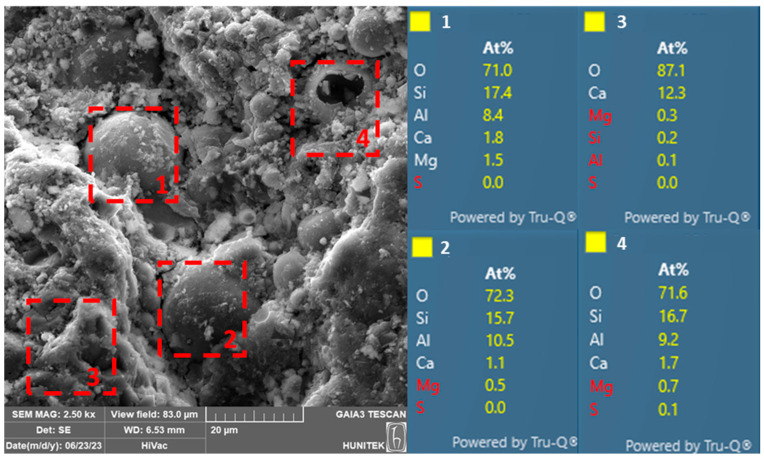
SEM/EDX of ECC-SSC-1.2F coded mixture after 120 d.

The SEM/EDX analysis results of select ECC-SSC-0F and ECC-SSC-1.2F mixtures at the end of 120 d of moist curing are presented in Figure 8 and Figure 9, respectively. The SEM image of the ECC-SSC-0F mixture depicted in Figure 8 indicates a remarkably dense, compact, and homogeneous matrix. Comparatively, the ECC-SSC-1.2F mixture exhibits a distinctively more heterogeneous and porous structure than the ECC-SSC-0F, in line with the reduced mechanical performance of the FA-based SSC-ECCs. Upon closer examination, numerous regions exhibited the presence of hydration products, as supported by the data from the EDX analysis. Indeed, the abundance of calcium, silicon, and aluminum in the matrix indicates the existence of C-S-H/C-A-S-H compounds as the main reaction products in the FA-based SSC-ECCs. In addition, the reaction products of ECC-SSC-0F shown in spectrums 2–4 presented higher Al/Ca ratios (around 0.3 in spectrum 3) as compared to the ECC-SSC-1.2F mixture (less than 0.01). This confirms the existence of lower ettringite formations in ECC-SSC-1.2F, as attributed to the incorporation of fly ash with a lower alkali content, thus negatively effecting the dissolution of the slag and FA particles [36]. The presence of high amounts of unreacted fly ash particles in Figure 9 also supports this explanation. In addition, greater Si/Ca ratios of 0.99–1.2 can be noticed in spectrums 2–4 of the ECC-SSC-0F than that of spectrum 3 in ECC-SSC-1.2F (almost 0.02). This indicates important reductions in the content of the C-S-H and C-A-S-H gels with the addition of FA to SSC cement [43], which supports the previous discussions for the decreased mechanical strengths at higher FA/SSC ratios.

### 3.7. XRD Analysis

**Figure 10:**
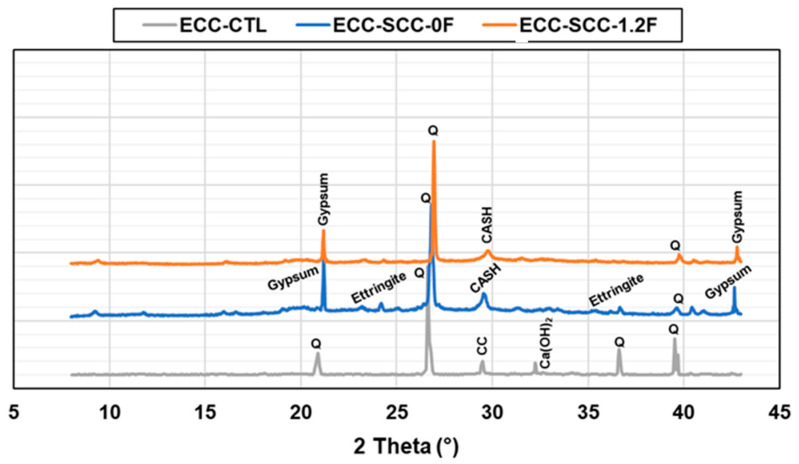
XRD analysis results of control (ECC-CTL) and SCC-based ECC-SSC-0F and ECC-SSC-1.2F mixtures after 120 days.

The XRD analysis results of the ECC-CTL, ECC without FA, and FA-based SSC-ECC mixtures at the end of 120 d are given in Figure 10. According to the results, the ECC-CTL mixture exhibited some silica/quartz, calcite, and calcium hydroxide peaks. ECC-SSC-0F mixture showed silica, CASH, ettringite, and gypsum peaks; whereas the ECC-SSC-1.2F mixture also showed peaks mainly related to silica and gypsum. As explained previously, it was confirmed in the XRD analysis that the formation of ettringite decreased with the increasing amount of fly ash, in line with the compressive and flexural strength results. In addition, the peak of gypsum was related directly to the SSC content, since higher gypsum phases were present in the ECC-SSC-0F than FA-based SSC-ECC [44]. Although, all three mixtures displayed peaks associated with silica, the intensity of this element at approximately 29° to 30° degrees 2θ was notably enhanced in ECC-SSC-1.2F, confirming a higher concentration of C-S-H/C-A-S-H gels within the system. This supports the previous discussions about the positive effect of FA in further increasing the chloride resistance of SSC-ECCs.

## 4. Conclusions

This paper investigated the performance of super-sulphated cement (SSC)-based ECC mixtures in comparison to ordinary Portland cement (OPC)-based ECC (ECC-OPC). In addition, the outcomes of fly ash (FA) addition at various FA amounts were also examined. The primary aim was to evaluate whether these environmentally friendly ECCs with SSC cement and varying FA content could meet the desired fresh, mechanical, and physical characteristics comparable to normal ECC. The findings of this research yield the following conclusions:

- All SSC-based ECCs demonstrated lower compressive and flexural strengths, as well as UPV results compared to the control ECC-CTL mixture. However, except for ECC-SSC-1.5F, which presented a lower strength than 30 MPa at 28 days, all the other SSC-based ECCs satisfied the requirement of most engineering applications, especially at FA/SSC ratios of 0 and 0.8.
- Different to ECC-SSC-0F, which demonstrated the lowest deformation amongst other ECCs, the deformation values of the FA-based SSC-ECCs highly improved to reach between 11.6% and 83.7% higher values than the ECC-CTL at 28 days.
- Negligible chloride ion permeability with extremely low RCPTs were found for all SSC-ECCs, especially at 0% FA.
- The use of SSC by OPC replacement caused the drying shrinkage to enhance dramatically, particularly at advanced curing ages. However, the inclusion of higher FA/SSC ratios at 1.2 and 1.5 resulted in important reductions in the shrinkage of ECC-CTL.
- The SEM analysis revealed that ECC-SSC-0F exhibited a dense and homogeneous matrix, while ECC-SSC-1.2F displayed a higher prevalence of ettringite structures due to the integration of FA with high aluminum content. The EDX data confirmed the presence of calcium–aluminum–silicate compounds in both mixtures, with ECC-SSC-1.2F having a higher concentration of silicon and a predominance of C-S-H and C-A-S-H hydration products.
- The XRD patterns confirmed the influence of FAs on the formation of ettringite phases in FA-based SSC-ECCs. However, the enhanced intensity of the silica peak supported the higher formation of C-S-H/C-A-S-H within the system.

These results confirm the possibility of completely replacing OPC with SSC, while achieving SSC-ECC compositions with increased sustainability and advanced ductility performance compared to conventional OPC-ECC. Moreover, according to the findings, the inclusion of SSC cement had minimal to negligible effects on the engineering applications of conventional ECCs. Therefore, all SSC-ECC mixtures, except those with an FA/OPC ratio of 1.5, can be safely employed in various structural and non-structural applications. These applications include building facades, architectural elements, bridge deck overlays, repair and rehabilitation projects, highway pavements, and marine structures, where moderate to high compressive strengths are required, alongside reduced permeability, enhanced crack resistance, and ductility. Furthermore, SSC-ECCs are suitable for use in hydraulic structures such as dams, spillways, and water-retaining structures, benefiting from ECC’s capacity to withstand hydraulic pressures, resist abrasion from flowing water, and endure chemical exposure.

## Figures and Tables

**Table 1:** Principal characteristics of OPC, slag, and fly ash.

Chemical Arrangement (%)	OPC	Slag	FA
Si	19.5	36.8	57
Al	5.1	8.7	21
Fe	2.9	0.6	4.2
Mg	2.5	11.0	1.8
Ca	61.8	38.1	9.8
Na	0.3	0.2	2.2
K	1.1	0.3	1.5
Loss on Ignition	2.5	1.1	1.3
SiO_2_ + Al_2_O_2_ + Fe_2_O_3_	27.5	46.1	82.2
Specific Gravity	3.15	3.1	2.38

**Table 2:** Compressive strengths of SSC mortars.

Mix ID	OPC (%)	Slag (%)	Gypsum (%)	Water to OPC	Sand to OPC	3-Day Compressive Strength (MPa)	7-Day Compressive Strength (MPa)
1	1	-	-	0.5	3	28.4	34.1
2	0.1	0.85	0.05	0.5	3	15.8	30.7
3	0.1	0.8	0.1	0.5	3	12.3	24.7
4	0.01	0.89	0.1	0.5	3	10.2	21.0

**Table 3:** Mix proportions of the mixtures (units are in kg/m^3^).

Mixture ID	OPC	Slag	Gypsum	FA	Sand	Water	Fiber	HRWRA
ECC-CTL	572	-	-	686	453	340	26	6
ECC-SSC-0F	130	1101	65	-	466	350	26	7
ECC-SSC-0.8F	69	586	34	552	447	335	26	6.5
ECC-SSC-1.2F	55.9	475	28	671	443	332	26	6
ECC-SSC-1.5F	49.1	417	24	735	441	331	26	5

## Data Availability

Data are contained within the article.
